# Resource efficient aortic distensibility calculation by end to end spatiotemporal learning of aortic lumen from multicentre multivendor multidisease CMR images

**DOI:** 10.1038/s41598-023-48986-6

**Published:** 2023-12-08

**Authors:** Tuan Aqeel Bohoran, Kelly S. Parke, Matthew P. M. Graham-Brown, Mitul Meisuria, Anvesha Singh, Joanne Wormleighton, David Adlam, Deepa Gopalan, Melanie J. Davies, Bryan Williams, Morris Brown, Gerry P. McCann, Archontis Giannakidis

**Affiliations:** 1 School of Science and Technology, Nottingham Trent University, Nottingham, NG11 8NS UK; 2 Department of Cardiovascular Sciences, University of Leicester and the NIHR Leicester Biomedical Research Centre, Glenfield Hospital, Leicester, LE3 9QP UK; 3 Imperial College London & Cambridge University Hospitals, Cambridge, CB2 0QQ UK; 4 Leicester Diabetes Centre, University of Leicester and the NIHR Leicester Biomedical Research Centre, Leicester General Hospital, Leicester, LE5 4PW UK; 5 Institute of Cardiovascular Science, University College London (UCL), National Institute for Health Research (NIHR), UCL Hospitals Biomedical Research Centre, London, WC1E 6DD UK; 6 Department of Clinical Pharmacology, William Harvey Research Institute, Queen Mary University of London, London, EC1M 6BQ UK

**Keywords:** Cardiology, Mathematics and computing

## Abstract

Aortic distensibility (AD) is important for the prognosis of multiple cardiovascular diseases. We propose a novel resource-efficient deep learning (DL) model, inspired by the bi-directional ConvLSTM U-Net with densely connected convolutions, to perform end-to-end hierarchical learning of the aorta from cine cardiovascular MRI towards streamlining AD quantification. Unlike current DL aortic segmentation approaches, our pipeline: (i) performs simultaneous spatio-temporal learning of the video input, (ii) combines the feature maps from the encoder and decoder using non-linear functions, and (iii) takes into account the high class imbalance. By using multi-centre multi-vendor data from a highly heterogeneous patient cohort, we demonstrate that the proposed method outperforms the state-of-the-art method in terms of accuracy and at the same time it consumes $\sim$ 3.9 times less fuel and generates $\sim$ 2.8 less carbon emissions. Our model could provide a valuable tool for exploring genome-wide associations of the AD with the cognitive performance in large-scale biomedical databases. By making energy usage and carbon emissions explicit, the presented work aligns with efforts to keep DL’s energy requirements and carbon cost in check. The improved resource efficiency of our pipeline might open up the more systematic DL-powered evaluation of the MRI-derived aortic stiffness.

## Introduction

### Clinical backdrop

The aorta is the main artery of the human body operating as a conduit that forwards oxygenated blood to peripheral organs and tissues. The elastic buffering capacity of the aortic wall is of vital importance to the cardiovascular system function as it caters to the transformation of blood flow from pulsatile (as generated by the left ventricular contraction) to steady (as required by the periphery)^1^. However, a progressive loss of the aortic wall elasticity manifests itself naturally with age^2^. Factors such as hypertension^3^, diabetes^4^, connective tissue disorders^5^, protein genetic variations^6^, and congenital heart defects^7,8^ may further escalate this process. The increased aortic stiffness has been shown to be an early marker of vascular aging and a powerful independent predictor of adverse cardiovascular events and mortality in various cohorts^9–13^.

Aortic distensibility (AD) is a direct measure of aortic stiffness and is defined as the maximum relative change in the aortic lumen cross-sectional area (A) during the cardiac cycle for a given pressure step at fixed vessel length

1 $$\begin{aligned} \text {AD}\, \big (10^{-3}\text {mmHg}^{-1}\big ) = \frac{A _{max }-A _{min }}{A _{min }\times PP } \end{aligned}$$

where PP is the pulse pressure (= systolic blood pressure − diastolic blood pressure), and A$_{max }$, A$_{min }$ denote the maximum and minimum areas, respectively^14,15^. AD is inversely proportional to the square of the pulse wave velocity^3^. Several reports^12,16–18^ have highlighted the value of AD as an indicator of aortic stiffness.

Cardiovascular magnetic resonance imaging (CMR) is recognised as the gold-standard non-invasive test for quantification of ventricular volumes and mass. Increasingly, CMR is used for the semantic segmentation-based calculation of AD from electrocardiogram-gated steady-state free precession (SSFP) cine images acquired in the plane perpendicular to the thoracic aorta at the level of the pulmonary artery bifurcation. This calculation in achieved by delineating the aortic lumen in the above 2D cine images, working out the minimum and maximum areas across the cardiac cycle by multiplying in each area the number of pixels by the pixel dimensions, and then combining those with a separately measured PP, as described in Eq. (1). Unlike other modalities used to assess aortic stiffness, CMR provides a high-resolution visualisation of the aorta in both spatial and temporal domains. Along with the reproducible placement of the imaging plane perpendicular to the vessel, CMR also permits the local stiffness evaluation at numerous aorta sections in the same study^3^. The CMR assessment of aortic stiffness has been validated against invasive intra-aortic pressure measurements^19^.

### The challenge

However, the image processing-based workflows for calculating AD from aortic cine CMR images are suboptimal in current clinical practice^20–22^, as they rely on semi-automated methods for the segmentation of the ascending aorta (AAo) and descending aorta (DAo) cross-sectional areas throughout the cardiac cycle. As well as being time-consuming, the image interpretation step of such pipelines is also subject to intra- and inter-observer variability. Additionally, the CMR expertise is costly. To enhance the clinical applicability of the CMR-derived AD and, thus, foster the efficient management of individuals with increased aortic wall stiffness, fast fully-automated methods, which also improve the robustness of the aortic lumen area quantification during the cardiac cycle, are needed. The automation to the cine CMR image interpretation analysis in the AD calculation is nonetheless challenging due to: (i) the high cross-sectional aortic shape variability across the cardiac cycle, different patients, and diverse pathologies, and (ii) the aorta brightness heterogeneity as a result of blood flow. In addition, the CMR acquisition protocols are very diverse among different studies and institutions which further adds to the task’s complexity^23^.

### Related work

Two recent papers deployed deep learning (DL)-based approaches to fully automate the aortic lumen segmentation from cine CMR images^24,25^. DL or hierarchical representations learning is a rapidly growing branch of machine learning in which the models learn complex raw-input-data representations that are tuned to the specific task at hand^26^. The deployment of DL models has been a game changer in computer vision (among a wide range of other industries) as it has permitted to achieve or even surpass human-level performance in a number of visual tasks including image classification, object detection, and semantic segmentation^27^. Recent research has highlighted the superb capabilities of DL models to analyse CMR images^28,29^. However, the two aorta DL studies treated the task at hand as a sparse annotation problem and evaluated their method only on a very limited number of cardiac cycle time frames (namely the end diastole (ED) and end systole (ES)) for which the ground truth labels were available. The targets of the remaining time frames (that were also used to map the input data during training) could not be visually validated and were acquired by pipelines which are likely to suffer from registration errors or poor convergence of the active contour algorithm. On top of this, the previous studies either completely ignored the temporal continuity inherent in the cine image sequence by treating the image segmentation task as static^25^, or simulated the use of time by stacking the recurrent part after the convolutional layers^24^. However, correlated spatio-temporal features cannot be learnt when spatial and temporal features are explicitly determined in separate regions of the network^30^. In addition, the previous papers combined the feature maps from the encoder and decoder using simple concatenation. Nonetheless, it has been argued that such a practice results in less precise segmentation than when non-linear functions are employed for this task^31^. Next, the loss function used by previous studies neglected the fact that the region of interest (ROI) class is significantly smaller than the background class. Moreover, the previous papers analysed datasets acquired by following a single data acquisition protocol on a relatively healthy cohort. Finally, previous work completely ignored the resource efficiency matter of the proposed pipelines. However, this is an important issue as the substantial computation and energy demands by DL models go together with a considerable environmental and financial cost^32^. Improving the efficiency of algorithms should be a high priority in DL research alongside accuracy^32^.

### Overview of the proposed method

In this paper, we propose to enhance the AD calculation by performing aortic lumen segmentation throughout the cardiac cycle using of a novel resource-efficient spatio-temporal DL model, inspired by the bi-directional ConvLSTM (BConvLSTM) U-Net with densely connected convolutions^31^. Our network is trained from scratch and evaluated on an aortic cine CMR dataset that was fully annotated by experts. The present paper is the first work to perform end-to-end (i.e., over the entire cardiac cycle) hierarchical learning and testing of the aortic lumen area from cine CMR images. Our approach joins the temporal with the spatial processing of the video input by merging the encoder and decoder feature maps through a BConvLSTM (non-linear) unit^33^. We employ the focal Tversky loss during training which is better suited for problems with a high class imbalance in the data^34^. We use multi-centre multi-vendor data from a highly heterogeneous patient cohort which significantly adds to the generalisation power of the proposed aortic lumen segmentation algorithm. Finally, the network we propose in this study is resource-efficient to help promote environmentally friendly and more inclusive DL research and practices. We perform quantitative evaluations of the energy consumption and carbon cost during training. It is worth noting that resource-efficient approaches are also more well-suited towards deployment in hardware-constrained platforms, which in turn will open up the more widespread DL-powered evaluation of the CMR-derived aortic stiffness. To examine the impact brought by each contributing factor, we perform ablation studies.

## Results

### Model accuracy

**Figure 1:**
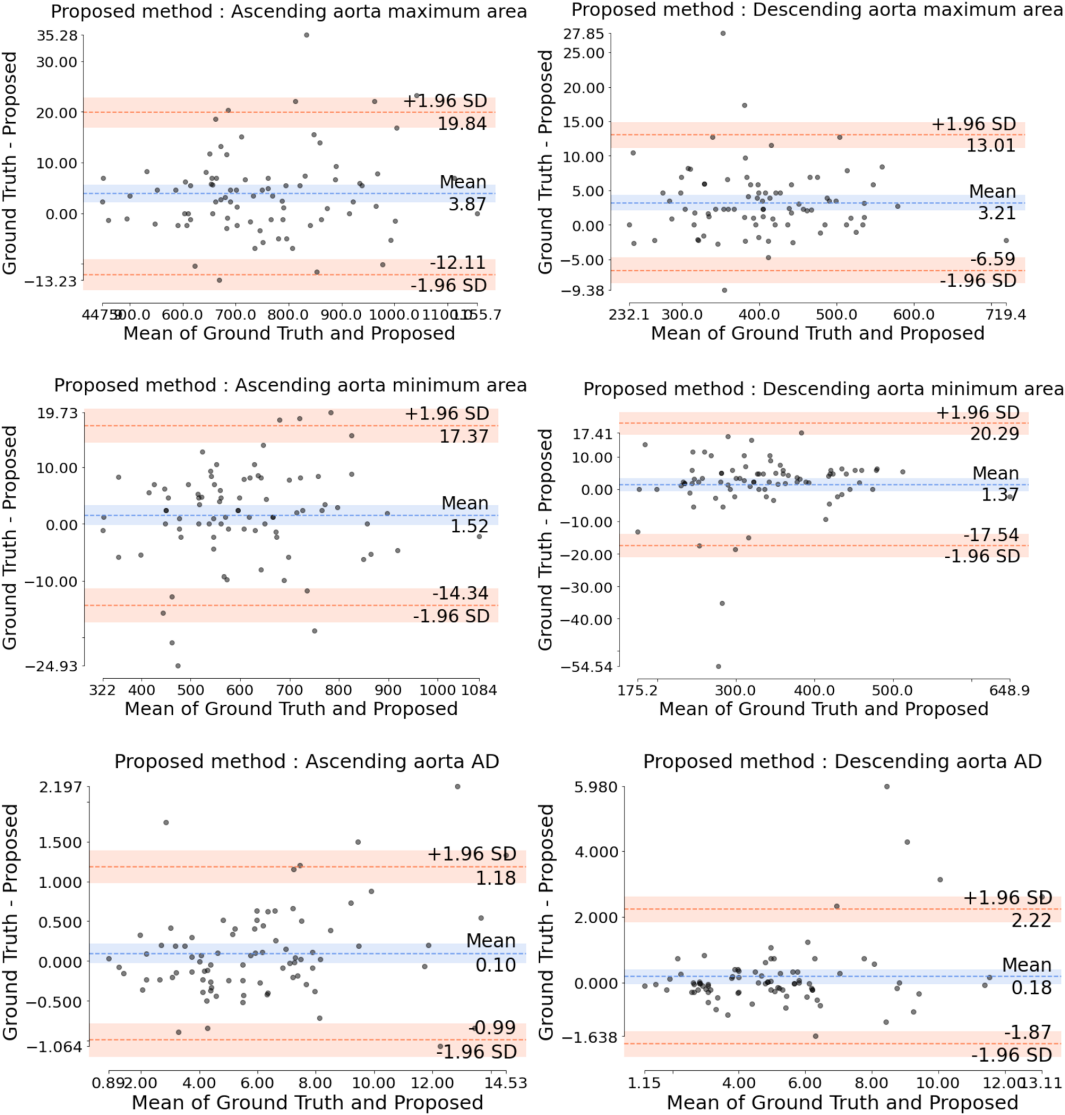
Bland-Altman analysis for graphically comparing the proposed method to the ground truth with respect to aorta maximum areas, aorta minimum areas and aortic distensibility (AD) values. Y-axis gives the difference between the two methods whereas X-axis represents their mean. Area is measured in mm${^2}$. AD is measured in 10$^{-3}\times$mmHg$^{-1}$. SD is the standard deviation.

**Figure 2:**
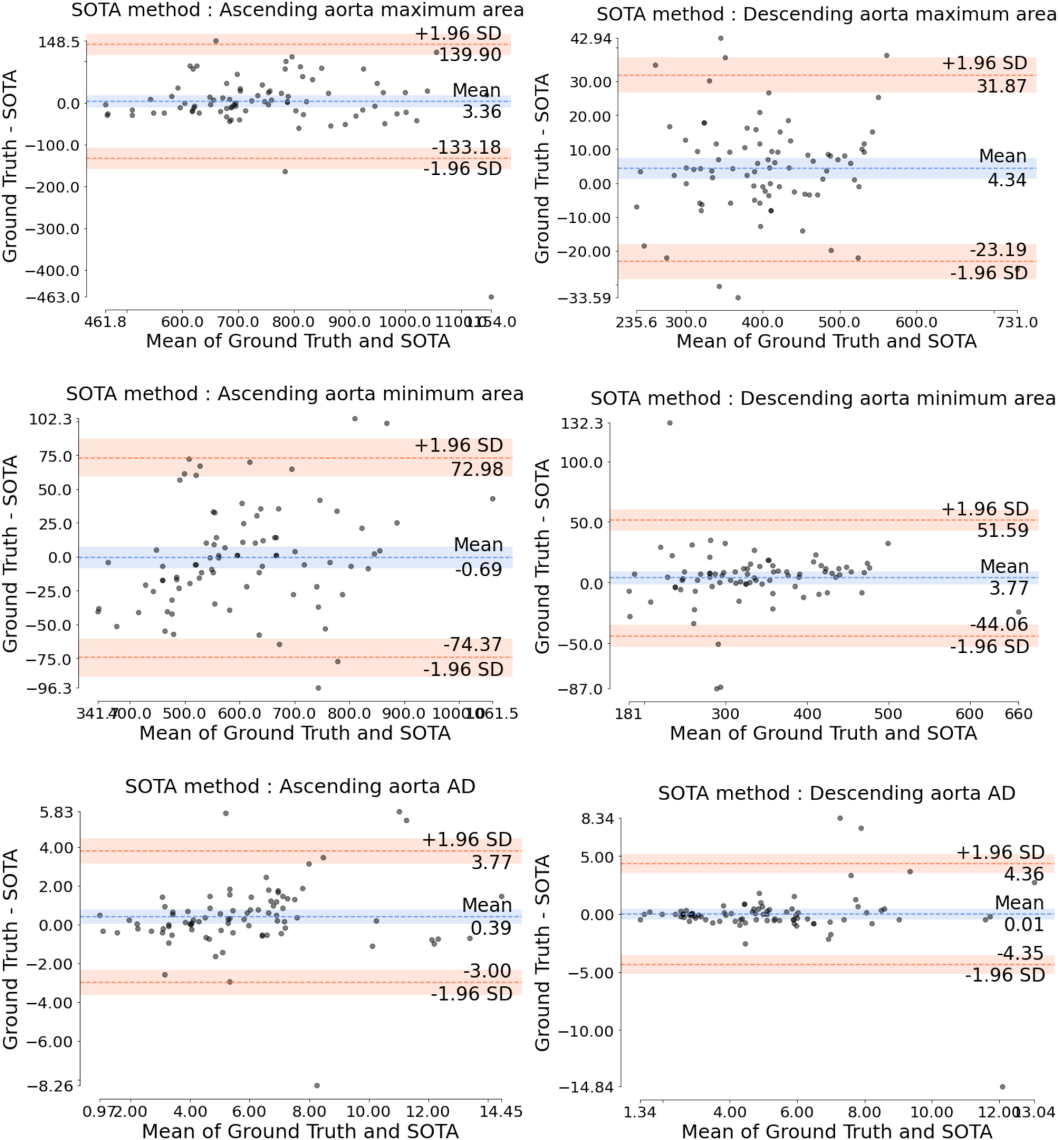
Bland-Altman analysis for graphically comparing the SOTA method^24^ to the ground truth with respect to aorta maximum areas, aorta minimum areas and aortic distensibility (AD) values. Y-axis gives the difference between the two methods whereas X-axis represents their mean. Area is measured in mm${^2}$. AD is measured in 10$^{-3}\times$mmHg$^{-1}$. SD is the standard deviation.

**Figure 3:**
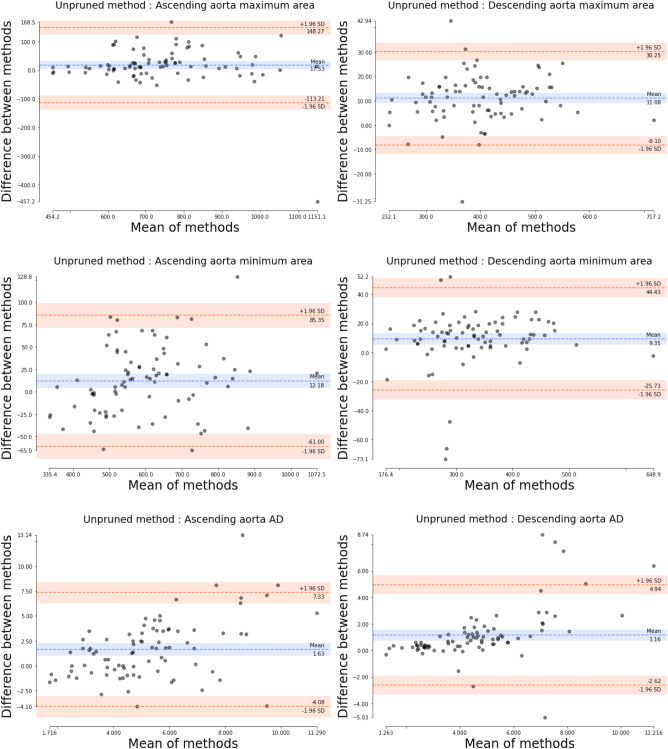
Bland-Altman analysis for graphically comparing the unpruned method^31^ to the ground truth with respect to aorta maximum areas, aorta minimum areas and aortic distensibility (AD) values. Y-axis gives the difference between the two methods whereas X-axis represents their mean. Area is measured in mm${^2}$. AD is measured in 10$^{-3}\times$mmHg$^{-1}$. SD is the standard deviation.

**Figure 4:**
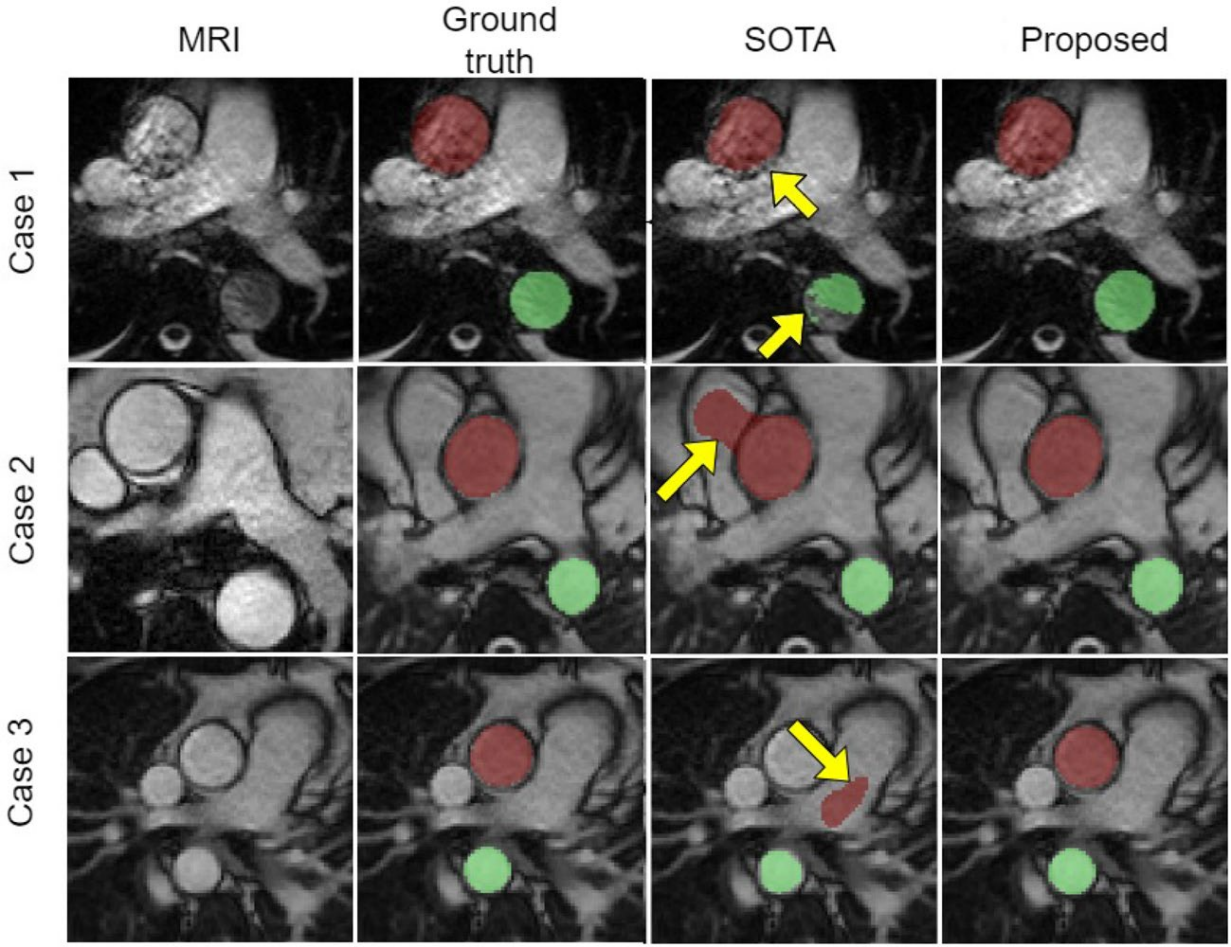
Qualitative comparison of the proposed method with the state-of-the-art (SOTA) method^24^ in one time frame of the cardiac cycle for three representative cases. First column: MRI. Second column: Ground truth (semi-automated segmentation). Third column: Segmentation results of the SOTA method^24^. Fourth column: Segmentation results of the proposed method. The yellow arrows indicate the errors in segmentation. The ascending aorta (AAo) is denoted by the red area and the descending aorta (DAo) is denoted by the green area.

Table 1 details the absolute errors in aortic area (in mm$^2$) and AD (in mmHg$^{-1}$) as well as the Dice coefficient for both AAo and DAo. The proposed method attained lower absolute area and AD errors and higher Dice coefficient values in comparison to both the state-of-the-art (SOTA) and unpruned methods. We have observed every model reaching a plateau before 250 epochs. Figures 1, 2 and 3 display the (maximum and minimum) area and AD Bland-Altman analysis plots for the proposed, SOTA and unpruned methods, respectively, versus the ground truth. It can be seen from the the limits of agreement of the Bland-Altman analysis plots versus the ground truth that the maximum and minimum aorta area as well as the AD values predicted by the proposed method had, on average, $\sim$ 3.9 times less fluctuation than the SOTA and unpruned methods. Figure 4 qualitatively compares the proposed and SOTA networks for three cases. Case 1 shows an instance where the SOTA method severely underestimated the number of pixels that had to be assigned to the aorta. Case 2 is a patient where the SOTA method over-segmented the AAo. Case 3 is a rare instance where the DAo is in an abnormal position relative to the AAo and for which the SOTA method was unable to identify the AAo. Our method accurately segmented both AAo and DAo for all three cases (Table 2).

Table 3 shows the results of the qualitative and quantitative comparisons of ascending and descending aorta time-area curves for one cardiac cycle of a representative case. It is apparent that the cardiac cycle phases of the proposed and the ground truth curves are in much closer agreement (for both AAo and DAo) than in the SOTA cases.

### Model resource efficiency

Table 2 gives the predicted carbon dioxide equivalent (CO$_2$eq) emissions (in g), the consumed energy (in kWh) and the equivalent distance (in km) a car could travel during the final training (250 epochs) for the proposed, SOTA and unpruned models. Also listed in Table 2 are the training time (in h:min:s) and the average inference time (in ms). The proposed method was $\sim$ 2.8 times more environmentally friendly and it consumed $\sim$ 3.9 times less fuel when compared with SOTA. In addition, it required $\sim$ 5.2 times less time to train, and its inference time was $\sim$ 2.7 times faster. Our method was also $\sim$ 5.2 times less polluting and it consumed $\sim$ 6.6 times less fuel when compared with the unpruned method. Lastly, it required $\sim$ 9.3 times less time to train, and its inference time was $\sim$ 4.4 times faster than the unpruned version.

### Ablation study

In this section, we carried out ablation experiments over a number of features of the proposed framework to better appreciate their proportionate significance. In particular:

*No full labels*: is trained using labels obtained by propagating ES and ED labels using non-rigid image registration.

*No focal Tversky*: is trained using Dice coefficient loss, ignoring the severe class imbalance in the data.

*No non-linearities*: is trained by merging the encoder and decoder feature maps through linear concatenation.

*No dense pruning*: is trained using three densely packed convolutional blocks in the final encoding step.

*No filter pruning*: is trained using four times more filters in the convolutional layers than the proposed model.

*No BConvLSTM pruning*: is trained using BConvLSTM unit in three steps instead of one.

To explore the effect of each factor on model accuracy, we employed the same evaluation metrics that were used in Table 1 and the same clinical images and hyperparameters that were used in the proposed framework. Table 4 gives the ablation results. It can be seen that not using non-linearities caused the largest drop in AD calculation performance. It is surprising that increasing the model size did not lead to accuracy improvement. This was possibly due to overfitting. In addition, not using focal Tversky loss and the full labels had more significant impact on the AAo AD calculation, most likely due to the increased number of false positives caused by surrounding structures. Lastly, it can be seen that the drop in performance for both the absolute error in area and Dice coefficient are large and uniform for every row in Table 4, whereas the absolute error in AD is a lot more variable. The reason for this is that the AD calculation is more susceptible to maximum and minimum area segmentation artefacts, while the segmentation accuracy metrics are averaged over the whole cardiac cycle, thus dampening the artefacts’ impact.

**Table 1:** Quantitative end-to-end performance of the proposed method using the absolute errors in aortic area and aortic distensibility (AD) and the Dice coefficient.

Model	Absolute error in area (mm$^2$)		Absolute error in AD (10$^{-3}\times$mmHg$^{-1}$)		Dice coefficient	
	AAo	DAo	AAo	DAo	AAo	DAo
**Proposed, mean (± SD)**	**7.346 (± 2.257)**	**4.749 (± 2.567)**	**0.394 (± 0.401)**	**0.544 (± 0.908)**	**0.989 (± 0.003)**	**0.991 (± 0.004)**
SOTA^24^, mean (± SD)	32.323 (± 25.420)	11.809 (± 10.204)	1.088 (± 1.395)	0.942 (± 2.013)	0.965 (± 0.0161)	0.978 (± 0.015)
Unpruned^31^, mean (± SD)	30.739 (± 21.110)	12.389 (± 7.209)	2.490 (± 2.218)	1.404 (± 1.759)	0.980 (± 0.009)	0.970 (± 0.012)
Proposed Vs SOTA^24^ *p* values	$1.710\times 10^{-15}$	$1.539\times 10^{-14}$	$5.291\times 10^{-08}$	$9.022\times 10^{-03}$	$1.710\times 10^{-15}$	$1.710\times 10^{-15}$
Proposed Vs Unpruned^31^ *p* values	$1.711\times 10^{-15}$	$3.503\times 10^{-15}$	$7.427\times 10^{-13}$	$4.888\times 10^{-07}$	$1.651\times 10^{-14}$	$1.779\times 10^{-15}$

Also provided are the *p* values obtained from the Wilcoxon-signed rank test with Bonferroni correction ($\alpha$ = 0.05). The mean ground truth area (averaged over time and patients) was 678.826 mm$^2$ (SD: 146.329 mm$^2$) for the AAo and 370.610 mm$^2$ (SD: 85.109 mm$^2$) for the DAo. Bold face indicates best performance.

AAo is the ascending aorta, DAo is the descending aorta, SD is the standard deviation and SOTA represents the state-of-the-art method^24^.

**Table 2:** Resource efficiency evaluation of the proposed method using the generated carbon emissions, the consumed energy and the equivalent distance a car could travel during the final training (250 epochs).

Model	CO$_2$eq (g)	Energy (kWh)	Equivalent distance travelled by car (km)	Training time (h:min:s)	Average inference time (ms)
**Proposed**	**2093.571**	**5.984**	**17.388**	**06:46:11**	**2.768**
SOTA^24^	5785.498	23.184	48.052	35:09:20	7.544
Unpruned^31^	11031.780	39.574	91.626	63:06:35	12.25

Also shown are the training and average inference times. The experiments were conducted on a workstation with an NVIDIA RTX A6000 (48GB) GPU. Bold face indicates best performance.

SOTA denotes the state-of-the-art method^24^.

**Table 3:** Qualitative and quantitative comparisons of ascending and descending aorta time-area curves for one cardiac cycle of a representative case.

Temporal curve	Fréchet distance	Hausdorff distance	Dynamic time warping (DTW) distance
	**16.822**	**16.822**	**165.424**
	37.384	37.384	337.390
	**5.607**	**4.673**	**63.552**
	9.346	6.542	85.048

SOTA denotes the state-of-the-art method^24^.

Bold face indicates best performance.

**Table 4:** Ablation over number of features using the proposed framework.

Model	Absolute error in area (mm$^2$)		Absolute error in AD (10$^{-3}\times$mmHg$^{-1}$)		Dice coefficient	
	AAo	DAo	AAo	DAo	AAo	DAo
**Proposed, mean (± SD)**	**7.346 (± 2.257)**	**4.749 (± 2.567)**	**0.394 (± 0.401)**	**0.544 (± 0.908)**	**0.989 (± 0.003)**	**0.991 (± 0.004)**
No full labels, mean (± SD)	29.964 (± 25.754)	8.649 (± 7.776)	4.017 (± 3.230)	3.038 (± 2.581)	0.983 (± 0.011)	0.970 (± 0.016)
No focal Tversky, mean (± SD)	30.034 (± 28.886)	10.399 (± 6.815)	25.499 (± 148.856)	2.161 (± 1.807)	0.981 (± 0.009)	0.969 (± 0.012)
No non-linearities, mean (± SD)	30.589 (± 20.173)	10.367 (± 7.943)	30.761 (± 210.163)	49.617 (± 437.580)	0.980 (± 0.016)	0.967 (± 0.0154)
No dense pruning, mean (± SD)	30.034 (± 28.886)	10.399 (± 6.815)	25.483 (± 148.859)	2.134 (± 1.86)	0.981 (± 0.008)	0.969 (± 0.012)
No filter pruning, mean (± SD)	28.966 (± 28.717)	9.322 (± 6.152)	10.901 (± 58.238)	1.991 (± 1.701)	0.982 (± 0.009)	0.971 (± 0.012)
No BConvLSTM pruning, mean (± SD)	46.811 (± 64.332)	17.288 (± 8.793)	46.502 (± 274.518)	2.116 (± 2.227)	0.971 (± 0.011)	0.962 (± 0.014)

“No full labels” is trained using labels obtained by propagating ES and ED labels using non-rigid image registration. “No focal Tversky” is trained using Dice coefficient loss, ignoring the severe class imbalance in the data. “No non-linearities” is trained by merging the encoder and decoder feature maps through linear concatenation. “No dense pruning” is trained using three densely packed convolutional blocks in the final encoding step. “No filter pruning” is trained using four times more filters in the convolutional layers than the proposed model. “No BConvLSTM pruning” is trained using BConvLSTM unit in three steps instead of one. Bold face indicates best performance.

### Ethical approval and consent to participate

Each study was approved by the UK national research and ethics service and written informed consent was obtained from all subjects prior to participation.

## Discussion

The elevated aortic stiffness has been linked to an increased risk of cardiovascular diseases. In this study, a novel resource-efficient DL model was proposed for the fast, robust, and fully automated segmentation of the AAo and the DAo from aortic cine CMR images towards streamlining quantification of AD.

### Strengths of this study

This study has a number of strengths compared to previous work. We resolved to not apply the semantic segmentation algorithm to each time frame of the cardiac cycle as a separate entity. Instead, we incorporated information related to both space and time into our task by merging the encoder and decoder feature maps in the second up-sampling layer through a non-linear function, namely a BConvLSTM. The use of the hyperbolic tangent function for combining the output of the forward and backward paths helps the network learn complex data structures^31^. To address the issue that the foreground is significantly smaller than the background class, we employed the focal Tversky loss during training. Following the enhanced credibility of the ground truth targets in all cardiac cycle time frames, we performed end-to-end hierarchical learning and testing of the aortic lumen area from cine CMR images, rather than focusing on a very limited range of time frames in the cardiac cycle. Another strength of this study is that we used multi-centre multi-vendor data from a highly diverse patient cohort which significantly adds to the ability of the proposed aortic lumen segmentation algorithm to generalise. The site, vendor, and patient heterogeneity when testing a model are crucial to the effective clinical implementation and to get approval by accreditation agencies. Unlike the model that motivated this paper^31^, our algorithm uses a building block that returns the sequence of feature maps over all time steps since we are dealing with video inputs. In addition, and in pursuit of a resource-efficient architecture, our algorithm: (i) has four times fewer filters in the convolutional layers, (ii) involves BConvLSTM layers in three times fewer steps, and (iii) contains only one third of densely packed convolutional blocks in the final encoding step.

### Main findings

The AAo and DAo segmentation masks predicted by our model were in close agreement with the semi-automated annotations by the CMR experts, as evidenced by the low area errors and the high Dice coefficient values. This finding corroborates that only one densely packed convolution block in the final layer of the contracting path sufficed to learn diverse features^35^. The proposed method was compared to other DL models that represent the SOTA and unpruned methods^24,31^. The quantitative analysis showed that our model outperformed both SOTA and unpruned methods in terms of segmentation accuracy, attaining lower aortic area and AD absolute errors and higher Dice coefficient values for both AAo and DAo. Furthermore, the limits of agreement of the Bland-Altman analysis plots versus the ground truth revealed that the maximum and minimum aorta area as well as the AD values predicted by the proposed method had, on average, $\sim$ 3.9 times less fluctuation than the SOTA and unpruned methods. These findings are in keeping with recent studies^36^, which reported that more powerful class-discriminative features can be captured in an efficient manner by tying up the temporal with the spatial processing in video inputs. Importantly and as an indication of an enhanced ability to generalise, our method, unlike SOTA , was found to accurately segment the aortic areas for a rare (and unseen by the algorithm) case for which the DAo was located at an abnormal position with respect to the AAo. Such an infrequent condition can be observed in individuals with scoliosis or patients with aortic arch branching and orientation abnormalities. Unlike previous studies^24,25^, the performance of the evaluated models on the AAo was slightly inferior to that on the DAo. A reason for this observation might be that the structures surrounding the AAo seem to be more challenging to differentiate between them in our diverse dataset. The consistency of the proposed segmentation method across the cardiac cycle was double-checked by demonstrating AAo and DAo time vs. area curves and estimating curve distances from the ground truth for a representative case. Finally, the proposed method was compared to SOTA and unpruned methods also in terms of resource-efficiency and carbon footprint using the Carbontracker tool^37^. Our method was found to consume $\sim$ 3.9 times less fuel and it was $\sim$ 2.8 less polluting during training than SOTA. In addition, it required $\sim$ 5.2 times less time to train, and its inference time was $\sim$ 2.7 times shorter than SOTA. As expected, our method was also $\sim$ 5.2 times less polluting and it consumed $\sim$ 6.6 times less fuel when compared with the unpruned method. Lastly, it required $\sim$ 9.3 times less time to train, and its inference time was $\sim$ 4.4 times faster than the unpruned version. Ablation studies showed that using BConvLSTM non-linearities gave the largest boost in AD calculation accuracy. Increasing the number of model parameters led to overfitting. Lastly, not using focal Tversky loss and the full labels had more significant impact on the AAo AD calculation, most likely due to the increased number of false positives caused by surrounding structures.

### Clinical implications

Previous experiments conducted in our lab have shown that the AD aortic stiffness measure has better reproducibility than the pulse wave velocity^38^. The excellent results presented in this paper allow to significantly reduce the time spent on extracting aortic structural and functional phenotypes from CMR data, while also improving the reliability of the results. Therefore, our pipeline could provide a valuable resource for exploring genome-wide associations of the AD and aortic areas with the cognitive performance for very large-scale biomedical databases (such as the UK Biobank)^39,40^, which better represent the wider population. Obtaining quantitative CMR phenotypes at such a scale remains a challenge nowadays. This type of analysis would enable us to investigate possible causal relationships of the aortic measures with aortic aneurysms and brain small vessel disease, as well the bidirectional relationship with blood pressure indices. The definition of the responsible mechanisms would eventually lead to (i) improved understanding of the factors that contribute to cognitive decline and dementia, and (ii) identification of new therapeutic targets. Moreover, the model developed for the AD quantification task could be reused as the starting point for various clinically relevant tasks (through transfer learning) to allow rapid progress and enhanced performance. The proposed framework could easily be integrated within a CMR analysis software. We have shared the code of our image analysis pipeline online (https://github.com/tuanaqeelbohoran/Aortic-Distensibility.git).

For our method to substantially impact routine clinical care, appropriate infrastructure for deployment and evaluation within the healthcare centre is needed. This ideally should include ML-ops platforms, pipelines for ensuring safety standards through identifying potential (such as methods for recognizing when the algorithm is operating in a more complex landscape than the one used for training or model explainability methods that highlight decision-relevant parts of feature representations).

### Resource efficiency considerations

It was found that the computations required for DL research have grown 300,000-fold from 2012 to 2018^41^, which is much faster than the rate at which compute demand has historically increased. These computations require staggering amounts of energy for fuelling them^32^. Given that electricity usage is correlated with greenhouse gas emissions, the associated carbon footprint is also outsized which appears to accelerate global climate change^32^. Even though the biggest part of this problem is caused by the very large-scale models used in natural language processing applications, the energy consumed by typical medical image analysis DL models is far from negligible. We estimated that the energy required to train our model was 5.984 KWh, as opposed to 23.183 KWh for the SOTA model. The respective generated carbon emissions were equivalent to those produced when a car travels a distance of 17.388 km for our model versus 48.052 km for SOTA. However, it is important to understand that those figures refer to the final training only. Before the final training run, resolving the optimal model in a standard development process necessitates multiple training runs due to the need for hyperparameter tuning and experimenting with various model architectures. To get a better handle on what the full DL model development pipeline might look like in terms of energy usage and carbon footprint, a recent study found that the process of building and testing a final paper-worthy DL model required training 4789 models over a 6-month period^42^. The significance of the dire numbers mentioned above is colossal, especially after taking the worldwide adoption of healthcare DL applications into consideration. Despite the fact that few concerns involving the energy usage and carbon footprint of DL research started to emerge^32,37,42^, the overwhelming majority of DL research in computer vision for healthcare are only concerned with enhancing accuracy while ignoring resource efficiency. In this paper, we made energy usage and CO$_2$eq emissions explicit. We endorse prior calls for making those key metrics evaluation criteria alongside accuracy-related measures in DL research, as a means to accelerate innovations in DL algorithmic efficiency^32,42–45^. The value of DL models should be judged by the amount of intelligence they provide per joule. Such an initiative would ultimately help: (i) reduce the adverse environmental impact of DL research during training and development, and (ii) make DL research more inclusive by letting more people participate^45^. With the spectre of an energy-hungry future looming alongside the escalating rates of natural disaster, it is imperative to explore ways to keep DL’s energy usage and carbon cost in check^32^. Finally, it is worth noting that the resource efficiency element is appealing also because it might increase the portability and practicality of our approach by promoting its wider distribution to devices with lower computational power and memory, which in turn might open up the more universal and systematic DL-powered evaluation of the CMR-derived aortic stiffness.

### Study limitations

This study has several limitations. Despite the large and diverse training dataset, our network is likely to produce less-accurate results if it is presented with pathologies, ages, ethnic backgrounds, and scanners that have not been included in the training set. In fact, this is the main limitation hampering the deployment of any DL model in the real world. To deal with this issue to a certain extent, we employed data augmentation techniques that simulate various possible data distributions. Altogether, it was illustrated that our pipeline exhibited improved ability to generalise to an unseen non-representative case compared to SOTA. Another main limitation of our model is that it lacks interpretability due to the intrinsic “black-box” nature of the DL algorithms. Explainable tools are a prerequisite for building trust in DL models, and the development of such a module will be a topic of future research^46^. Furthermore, our model has not been tested against adversarial attacks^47^. Even though Carbontracker supports a variety of different environments and platforms, there might be small deviations of the reported energy consumption and carbon emission values from the true ones. Such deviations might be caused by the quality of the estimated carbon intensity of electricity production which varies by geographic location (depending on the energy sources that power the local electrical grid) and time throughout the day (as energy demand and capacity change). However, all DL experiments in this study were conducted at the same workstation and time period during the day. In addition, Carbontracker uses “real-time” carbon intensity values which are fetched every 15 min during training using the application programming interfaces supported by this tool. In this study, the predicted maximum and minimum aortic areas, that were also used for the quantification of AD, did not always relate with the diastolic and systolic cardiac cycle phases, respectively, of the handcrafted analysis. However, this did not significantly affect the AD measurements. Our study required full annotation of all temporal frames in the cine dataset, as opposed to SOTA that required only sparse annotation. However, the SOTA method also needs extra resources (people, time) to identify ED and ES phases. It also needs significant resources (hardware, time) to perform the very computationally-intensive non-rigid registration. Lastly, the annotation in our study was not so time-consuming because it was largely supported by the JIM software that performed automated label propagation, which was then verified by the clinicians. Therefore, the consensus on the standards is that our study is concerned only with training resources. Finally, other recent fully-automated segmentation approaches do exist^48,49^, that could potentially perform better than ours in the aortic lumen delineation task from cine CMR images. However, the goal of this work was to propose a DL-based framework that surpasses the SOTA methods for this particular task while being more resource-efficient, rather than to carry out an exhaustive survey of semantic segmentation, the literature of which is huge.

## Conclusion

This paper proposed a novel resource-efficient DL model for the fast, robust, and fully-automated segmentation of the AAo and DAo from aortic cine CMR images towards streamlining quantification of AD. When evaluated on a large multi-centre multi-vendor dataset from a highly heterogeneous patient cohort, the proposed method outperformed the SOTA method in terms of accuracy and at the same time it consumed $\sim$ 3.9 times less fuel and generated $\sim$ 2.8 less carbon emissions. Notably, the proposed method was even more accurate than the unpruned method. Our model could provide a valuable tool for exploring genome-wide associations of the AD and aortic areas with the cognitive performance in very large-scale biomedical databases. By making energy usage and greenhouse gas emissions explicit, the presented work aligns with efforts to keep DL’s energy requirements and carbon cost in check. The improved resource efficiency element of our pipeline might open up the more universal and systematic DL-powered evaluation of the CMR-derived aortic stiffness.

## Methods

### Study population and image dataset

The study population comprises participants from four clinical studies analysed at the University Hospitals of Leicester NHS Trust MRI core lab which included AD assessment. These included participants with spontaneous coronary artery dissection^50^, asymptomatic type 2 diabetes (from Lydia^51^ and DIASTOLIC^52^ trials), hypertension (the Pathway 2 study) and healthy volunteers (recruited in above studies^50,51^). In total, we analysed 424 aortic MRI datasets taken from 376 patients. The demographic, anthropometric and clinical characteristics of the participants are presented in Table 5. Each study was approved by the UK national research and ethics service and written informed consent was obtained from all subjects prior to participation. All methods were performed in accordance with the relevant guidelines and regulations.

**Table 5:** Patient characteristics. Abbreviations: BMI, body mass index; SBP, systolic blood pressure; DBP, diastolic blood pressure; CVA, cerebrovascular accident; DM, type 2 diabetes mellitus.

	(n = 376)
Age, mean (± SD), y	48 (± 8)
Male, No. (%)	150 (40)
Female, No. (%)	226 (60)
BMI, mean (± SD), kg/m$^2$	31.74 (± 7.41)
SBP, mean (± SD), mmHg	127.33 (± 17.44)
DBP, mean (± SD), mmHg	79 (± 12.59)
Hypertension, No. (%)	197 (52)
Smoking, No. (%)	192 (51)
History of CVA, No. (%)	2 (1)
History of DM, No. (%)	208 (55)
Renal impairment, No. (%)	78 (21)

### MRI acquisition

Acquisitions were undertaken on either 1.5T or 3T scanners from three centres using four different MRI scanners: Leicester (Siemens Aera, 1.5T and Skyra, 3T), Cambridge (GE Signa, 1.5T) and Dundee (Siemens TrioTim, 3T). SSFP cine images of the AAo and DAo in a plane perpendicular to the thoracic aorta at the level of the pulmonary artery bifurcation were reconstructed to 30-40 phases as previously described^20,38^. The typical image matrix was 256 × 186 to 256 pixels. The in-plane pixel height and width varied between 1.093mm and 1.914mm. The slice thickness for cine imaging was 8mm. Brachial blood pressure was measured simultaneously to determine pulse pressure.

### Data pre-processing and annotation

The end-to-end data annotation was carried out semi-automatically by experts from the Glenfield hospital in Leicester using the Java Image Manipulation Software Version 6 (Xinapse Systems Ltd, Essex, UK)^53^ as previously described^20,38^. The ascending and descending aorta was analysed by manually contouring the first and last phase and every 6th phase in between. Then images were propagated to fill in the other phases of the sequence. All phases were then manually checked and adjusted if required. The experts were blinded to the patient details. All MRI images and masked images (annotations) were zero-padded to 256 × 256 pixels so that their dimensions match.

### Neural network architecture

**Figure 5:**
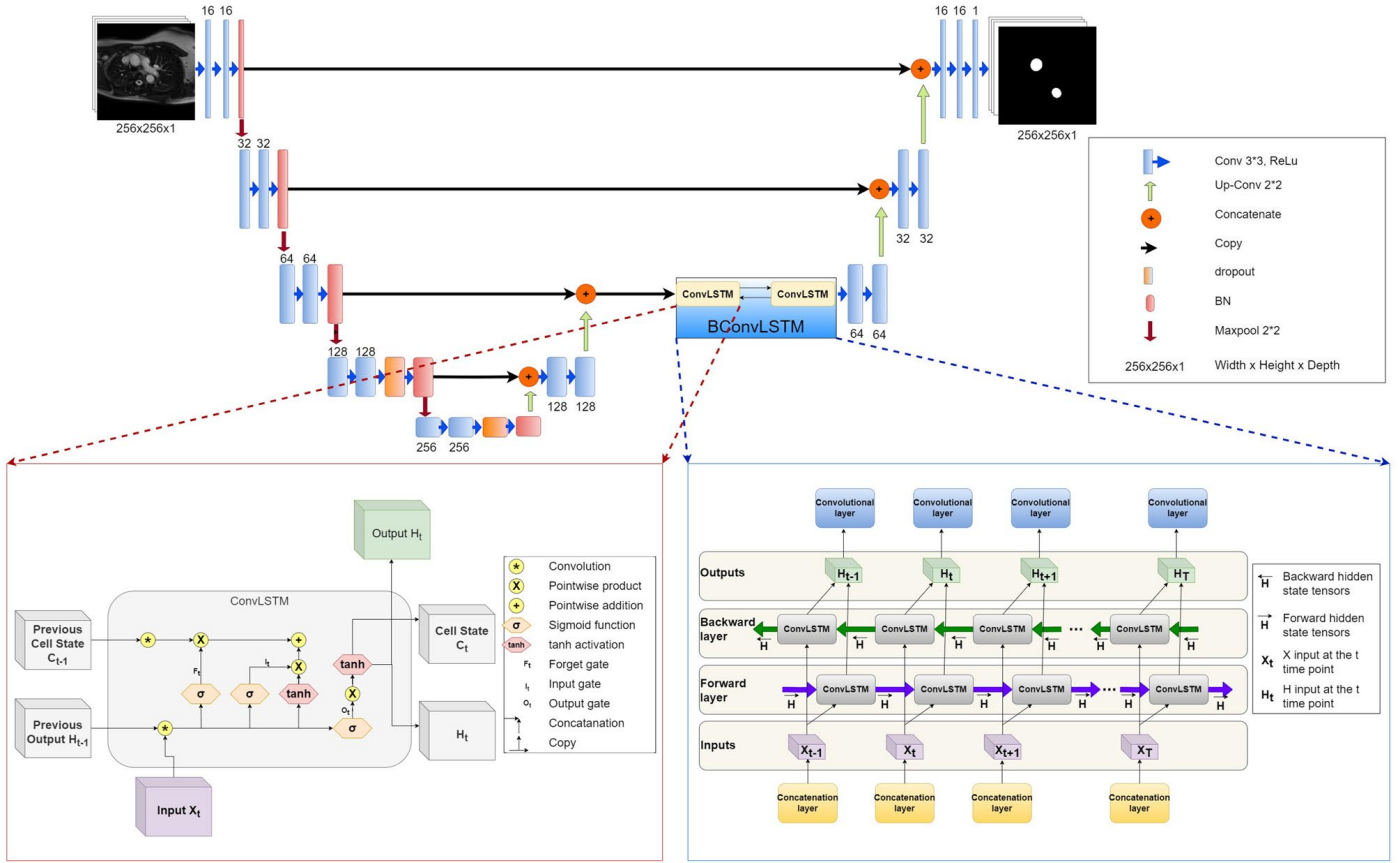
The proposed deep learning model, including two diagrams that illustrate how the temporal dimension is handled.

The proposed architecture (Fig. 5) was inspired by the BConvLSTM U-Net with densely connected convolutions^31^. It consists of: (i) a contracting path (a.k.a. the encoder) for capturing the context in the image by transforming it into a high-level feature representation, and (ii) a symmetric expanding path (a.k.a. the decoder) for interpreting the feature maps, enabling precise localisation (where in the image) and producing a full resolution segmentation map. There are four down-sampling layers in the encoder and four up-sampling layers in the decoder.

Each encoding step consists of a sequence of two convolutional layers (3 × 3 filters and a ReLU non-linear activation^54^) followed by a Batch Normalisation (BN) layer^55^ and a 2 × 2 max-pooling. BN was employed so that the optimiser converges faster when training the network, whereas the max-pooling operation was used to aggressively down-sample the feature maps. To reduce over-fitting, dropout regularisation^56^ was employed in the last two steps of the contracting path before the BN layer. The number of filters that each encoding layer computes over the input doubles at each step ([16, 32, 64, 128, 256]).

Each decoding layer consists of a sequence of two convolutional layers (3 × 3 filters and a ReLU non-linear activation) apart from the final decoding step that has five convolutional layers. In each step of the expanding path, the output of the previous layer is passed onto an up-conv layer (i.e. up-sampling function followed by a 2 × 2 convolution; this process doubles the size of the feature map) and then combined with the corresponding (same-resolution-level) representation in the contracting path using skip connections. The combination of these two types of feature maps is a channel-wise concatenation in all steps except for the second up-sampling layer, where we propose to merge them in a more complex way using (apart from concatenation also) a BConvLSTM^33^ building block that outputs information about all temporal hidden states. This is to account for the spatiotemporal composition of the input. The BConvLSTM building block is made of two ConvLSTMs. ConvLSTM is a variant of the traditional LSTM neural network that is specifically designed to process spatial data. By incorporating convolutional structures within the LSTM gates, ConvLSTM is capable of capturing spatial dependencies in the data. Figure 5 provides a visual representation of the ConvLSTM block operation. The ConvLSTM block’s distinctiveness lies in its ability to apply convolutional operations within the gates, enabling the capture of spatial dependencies in the data. Then, the BConvLSTM output $Y_j$ at the time step *j* is calculated as

2 $$\begin{aligned} Y_j = \text {tanh}(W_y^{\overrightarrow{H}} *\overrightarrow{H}_j + W_y^{\overleftarrow{H}} \overleftarrow{H}_j) +b, \end{aligned}$$

where $\overrightarrow{H}$ and $\overleftarrow{H}$ denote forward and backward hidden state tensors^31^, respectively, $W_y^{\overrightarrow{H}}$ and $W_y^{\overleftarrow{H}}$ denote the forward and backward convolution kernels corresponding to the hidden states, *b* represents the bias term, and the hyperbolic tangent was employed to combine the outputs of the forward and backward paths in a non-linear way. The mathematical equations for obtaining each of the two hidden state tensors are as follows:

3 $$\begin{aligned} I_t= & {} \sigma (W_{XI} * X_t + W_{HI} * H_{t-1} + b_I) \end{aligned}$$

4 $$\begin{aligned} F_t= & {} \sigma (W_{XF} * X_t + W_{HF} * H_{t-1} + b_F) \end{aligned}$$

5 $$\begin{aligned} C_t= & {} F_t \circ C_{t-1} + I_t \circ \tanh (W_{XC} * X_t + W_{HC} * H_{t-1} + b_C) \end{aligned}$$

6 $$\begin{aligned} O_t= & {} \sigma (W_{XO} * X_t + W_{HO} * H_{t-1} + b_O) \end{aligned}$$

7 $$\begin{aligned} H_t= & {} O_t \circ \tanh (C_t) \end{aligned}$$

Here, $I_t$, $F_t$, and $O_t$ denote the input, forget, and output gates, respectively, at time $t$, while $C_t$ and $\sigma$ represent the cell state and the sigmoid function, respectively, and $x_t$ refers to the input at time $t$ (Fig. 5). The input gate governs the information that is retained in the cell state. The forget gate regulates the information that is discarded from the cell state. The output gate controls the information utilized to compute the output of the LSTM. The cell state maintains the internal state of the ConvLSTM. The $*\text { operator}$ symbolises the convolution operation, and $\circ$ represents the Hadamard product (element-wise multiplication).

The number of channels reduces in every step of the expanding path ([256, 128, 64, 32, 16, 1]), whereas the size of the feature maps progressively increases to reach the input size after the final layer.

Unlike the Azad et al.^31^ that inspired this work, our method uses a BConvLSTM building block that returns the sequence of feature maps over all time steps since the dataset was time distributed. In addition, and in pursuit of a more efficient architecture, our method: (i) uses four times less filters in the convolutional layers compared to Azad et.al.^31^, (ii) involves BConvLSTM in only one step, (iii) contains only one densely packed convolutional block in the final encoding step.

### Implementation and training

The dataset was randomly split into a training set (272 datasets), validation set (68 datasets) and a testing set (84 datasets). For training, we used the Adam optimiser^57^ for 250 epochs with a constant learning rate of 0.001 and a batch size of 120 (approximately 4 patients). The dropout value that we used was 0.5. The initialiser of the network was He Normal^58^. In order to improve the proposed model’s ability to generalise, online data augmentation techniques were employed. The augmented data were obtained from the original images by applying random rotations (by a degree between -30$^{\circ }$ and +30$^{\circ }$) and random translations along the x- and/or y-axis in either direction (by up to 20 pixels). All hyperparameters were tuned using grid search based on the validation accuracy. To address the severe class imbalance between pixel values 0 and 1 in each frame, we utilised the Focal Tversky loss function defined as

8 $$\begin{aligned} \text {Focal Tversky Loss} = (1-\text {Tversky Loss})^{\gamma } \end{aligned}$$

where $\gamma$ is the adjustable focussing parameter and Tversky Loss is the Tversky index given by

9 $$\begin{aligned} \text {Tversky Loss} = \frac{\sum _{i}^{N} p_{0i} g_{0i} }{\sum _{i}^{N} p_{0i}g_{0i} + \alpha \sum _{i}^{N} p_{0i}g_{1i}+ \beta \sum _{i}^{N} p_{1i}g_{0i}} \end{aligned}$$

where $p_{0i}$ is the probability that pixel *i* belongs to the aorta and $p_{1i}$ is the probability of pixel *i* being in the background class^59,60^. In addition, $g_{0i}$ is 1 for the aortic vessel segmentation area and 0 for the background, and the opposite is true for $g_{1i}$. Finally, $\alpha$ and $\beta$ are variables in Tversky Loss, which control the magnitude of the penalties for false positives and false negatives, respectively. To improve model convergence and the recall rate^59,60^, we trained our model with $\alpha$ = 0.8, $\beta$ = 0.8 and $\gamma$ = 1. All the methods were trained using the TensorFlow environment.

### Model evaluation and statistical analysis

For evaluating the automated segmentation masks produced by our method relatively to the ground truth, we employed the Dice coefficient as well as the absolute area error (in mm$^2$) and absolute AD error (in mmHg$^{-1}$). In addition, we used Bland-Altman analysis for assessing the agreement between (maximum and minimum) aorta areas and AD values^61^. The temporal fidelity of the segmentation performance across a cardiac cycle was assessed both qualitatively and quantitatively using the Fréchet, Hausdorff and dynamic time warping (DTW) distances for a representative case. To examine the impact brought by each contributing factor, we performed ablation studies. All analyses described above was performed for both the AAo and DAo. The reported results refer to the test set.

For evaluating the resource efficiency of our method, we calculated the CO$_2$eq emissions (in g) and energy spent (in kWh) during training using the Carbontracker method^37^. Carbontracker is an open-source tool written in Python for tracking and predicting the energy consumption and carbon emissions of training DL models for a given GPU. To put the carbon footprint produced during model training in context, we also reported the equivalent distance travelled by car^62^ that would generate the same emission volume. The training and inference times were also computed. For comparison purposes, all evaluation metrics of the proposed method were juxtaposed with those produced by the SOTA^24^ and unpruned^31^ methods, also trained on the same multi-centre, multi-vendor multi-disease CMR dataset following similar training and hyperparameter tuning procedures as those described above. To determine if there is a statistically significant difference (at level 0.05) between the performances of the proposed and SOTA methods, we used the Wilcoxon signed-rank test with Bonferroni correction.

## Data Availability

The datasets generated and/or analysed during the current study are not publicly available due to privacy/ethical restrictions. If someone wants to request the data from this study, they should contact Prof. Gerry P. McCann at gpm12@leicester.ac.uk.

## Code availability

The link to the GitHub repository containing the code of the image analysis pipeline is: https://github.com/tuanaqeelbohoran/Aortic-Distensibility.git.
